# Cardiovascular Comorbidities in COVID-19: Comprehensive Analysis of Key Topics

**DOI:** 10.2196/55699

**Published:** 2024-07-24

**Authors:** Rene Markovič, Luka Ternar, Tim Trstenjak, Marko Marhl, Vladimir Grubelnik

**Affiliations:** 1 Faculty of Natural Sciences and Mathematics University of Maribor Maribor Slovenia; 2 Faculty of Electrical Engineering and Computer Science University of Maribor Maribor Slovenia; 3 Faculty of Medicine University of Maribor Maribor Slovenia; 4 Faculty of Education University of Maribor Maribor Slovenia

**Keywords:** COVID-19, cardiovascular diseases, metabolic disorders, embolism and thrombosis, hypertension, hyperglycemia, iron metabolism disorders, MeSH, embolism, thrombosis, heart failure, nutritional, vascular disease, glucose, effective

## Abstract

**Background:**

The interrelation between COVID-19 and various cardiovascular and metabolic disorders has been a critical area of study. There is a growing need to understand how comorbidities such as cardiovascular diseases (CVDs) and metabolic disorders affect the risk and severity of COVID-19.

**Objective:**

The objective of this study is to systematically analyze the association between COVID-19 and cardiovascular and metabolic disorders. The focus is on comorbidity, examining the roles of CVDs such as embolism, thrombosis, hypertension, and heart failure, as well as metabolic disorders such as disorders of glucose and iron metabolism.

**Methods:**

Our study involved a systematic search in PubMed for literature published from 2000 to 2022. We established 2 databases: one for COVID-19–related articles and another for CVD-related articles, ensuring all were peer-reviewed. In terms of data analysis, statistical methods were applied to compare the frequency and relevance of MeSH (Medical Subject Headings) terms between the 2 databases. This involved analyzing the differences and ratios in the usage of these terms and employing statistical tests to determine their significance in relation to key CVDs within the COVID-19 research context.

**Results:**

The study revealed that “Cardiovascular Diseases” and “Nutritional and Metabolic Diseases” were highly relevant as level 1 Medical Subject Headings descriptors in COVID-19 comorbidity research. Detailed analysis at level 2 and level 3 showed “Vascular Disease” and “Heart Disease” as prominent descriptors under CVDs. Significantly, “Glucose Metabolism Disorders” were frequently associated with COVID-19 comorbidities such as embolism, thrombosis, and heart failure. Furthermore, iron deficiency (ID) was notably different in its occurrence between COVID-19 and CVD articles, underlining its significance in the context of COVID-19 comorbidities. Statistical analysis underscored these differences, highlighting the importance of both glucose and iron metabolism disorders in COVID-19 research.

**Conclusions:**

This work lays the foundation for future research that utilizes a knowledge-based approach to elucidate the intricate relationships between these conditions, aiming to develop more effective health care strategies and interventions in the face of ongoing pandemic challenges.

## Introduction

The SARS-CoV-2 virus, which causes the disease COVID-19, has impacted all areas of our lives. The scientific community has shown an unprecedented and coordinated response to this global pandemic [1,2]. This has led to a rapid acquisition of new knowledge in a wide range of scientific fields and, simultaneously, to new questions that needed to be answered [3-5]. Some of the most important questions concern the origins and causes leading to the severe form of COVID-19 or even death [6,7]. One of the most important pathological aspects of COVID-19 disease is its impact on the cardiovascular system, more specifically cardiovascular disease (CVD) [8-11]. The link between COVID-19 and CVD has been demonstrated and confirmed in numerous studies. A recent scientific review article by Vosko et al [12] offers an extensive overview of the literature on the interaction between COVID-19 and CVDs. The authors describe how COVID-19 can act as a yet unrecognized risk modifier for CVD, including risk factors such as diabetes mellitus [13] or arterial hypertension [14]. In the study by Vosko et al [12], an increased incidence of CVD and poorer clinical outcomes were observed in individuals with preexisting CVD, noting conditions like myocarditis, acute coronary syndrome, heart failure, thromboembolic complications, and arrhythmias. Furthermore, the article by Vosko et al [12] summarizes the mechanisms through which COVID-19 can affect CVD, including the impact on endothelial cells and inflammation, which can increase the risk for atherosclerosis and other cardiovascular events. Additionally, a review study [15] was conducted to demonstrate the connection between COVID-19 and CVD. This study provides a detailed examination of the impact of COVID-19 on different cells in myocardial tissue and offers an overview of the clinical manifestations of cardiovascular involvement in the pandemic.

The most striking link between COVID-19 and CVD involves the angiotensin-converting enzyme 2 (ACE2), which is the main receptor for the glycoprotein membrane spike of SARS-CoV-2 [16-18]. ACE2 is bound to cell membranes in various tissues of the vascular system [19]. Considering its importance in CVD, a population-based study showed that higher ACE2 plasma levels are associated with a greater risk of severe CVD [20]. COVID-19 has been found to increase the risk of cardiogenic shock [21,22], cardiac arrhythmias [23,24], acute myocardial injury [25,26], and sometimes sudden death in patients with CVD [15,27,28], and at the same time, patients with CVD have a higher risk of mortality due to COVID-19.

ACE2 is an important down-regulator of the renin-angiotensin-aldosterone system (RAAS), which plays a significant role in controlling arterial blood pressure [29]. Various studies have investigated the dysregulation of ACE2 in different cells of patients with CVD, indicating an involvement of the RAAS [30,31]. For example, downregulation was found primarily in fibroblasts and the vascular smooth muscle of ventricles with dilated or hypertrophic cardiomyopathy [32,33]. Conversely, an upregulation of ACE2 is mainly observed in the cardiomyocytes of patients with ischemic and non-ischemic cardiomyopathy [32-34], It is also noted in the lungs of patients with hypertension, cerebrovascular disease, coronary artery disease, and other comorbidities such as diabetes [35], which may be attributable to the joint treatment of such comorbidities in addition to the disease itself [36].

This correlation is further supported by biochemical and genetic analyses, as patients with heart failure show increased ACE2 expression. ACE is found in 7.6% of all heart cells, compared to only 5.88% in healthy individuals. This is even more pronounced in cardiomyocytes, where 9.87% of all cardiomyocytes in heart failure express ACE2, whereas in healthy hearts, the figure is 6.75% of cardiomyocytes. This is reversed in arterial vascular cells: heart failure shows positive ACE2 expression in 7.93% of vascular cells and 19.4% in healthy individuals [37]. The invasion of SARS-CoV-2 upregulates the activity of the protease ADAM17, which in turn downregulates ACE2 by cleaving it from the cell surface. This process, known as “shedding,” and is very important for understanding the cardiovascular effects of COVID-19. Recognizing the beneficial effects of Ang-1-7 signaling, we understand that disruption of this pathway through shedding leads to the predominance of the RAAS, causing hypertension, fibrotic remodeling, inflammation, and sodium retention [38,39].

Novel big data streams have created interesting opportunities to synthesize research and identify hotspots of big data in infectious disease epidemiology [40]. Furthermore, big data bibliometric analyses can reveal trends and project future developments in each scientific discipline [41-43]. Thus, based on bibliometric analysis, a study was conducted [44], that aimed to investigate the international scientific output on the relationship between COVID-19 and CVDs. The findings revealed that the United States and China are at the forefront in both the quantity and quality of publications in this area. Additionally, the analysis indicated that researchers have paid special attention to cardiovascular comorbidities, outcomes, and regenerative medicine in the context of COVID-19. Such innovative analytical approaches, which leverage extensive big data resources, are particularly crucial for deciphering the complex dynamics of comorbidity patterns observed in COVID-19 and CVDs. By integrating big data insights with traditional epidemiological methods, our study not only contributes to a deeper understanding of these comorbidities but also opens new avenues for predictive analytics in health care.

Considering all this evidence, a critical interface between the virus and CVD has emerged, posing unique challenges to health care systems worldwide. This study aims to unravel the complex relationship between COVID-19 and CVD, addressing a significant gap in our current understanding of the comorbidity dynamics of these diseases. Utilizing a novel approach with MeSH (Medical Subject Headings) descriptors, we systematically analyze a wide range of literature to identify key patterns and themes. Our study not only sheds light on the increased risks and outcomes associated with these comorbidities, but also paves the way for future research methods. This manuscript is organized to first explain our methodological approach, followed by a presentation of our findings, a discussion of their implications, and concludes with insights that have the potential to inform future health care strategies and interventions.

## Methods

### Overview

We conducted a search on PubMed [45] with specific search queries on CVD and SARS-CoV-2 and limited our search to articles published from the year 2000 onwards. Between January 1, 2000, and September 30, 2021, we collected all relevant entries in PubMed. From these entries, we selected only peer-reviewed scientific publications. We then created 2 databases: one for articles related to COVID-19 (the COVID-19 database) and another for articles related to CVDs (the CVD database). The databases had a similar organization and stored 2 primary pieces of information: the PubMed identifier (PMID) and all the MeSH descriptors provided for an article. The following sections describe in detail the creation of the databases, the use of the MeSH classification scheme, and the analyses performed.

### MeSH Classification

The MeSH thesaurus is a controlled and hierarchically organized vocabulary developed and curated by the National Library of Medicine (NLM). The assignment of MeSH descriptors to papers by professional indexers at the NLM is highly consistent and an efficient method for describing the main topics of an article. Consequently, the MeSH classification system offers an organized approach for sorting and accessing medical knowledge. This knowledge is represented by MeSH descriptors or MeSH terms, which are organized hierarchically to facilitate efficient retrieval of biomedical and health-related information from the NLM databases.

**Figure 1:**
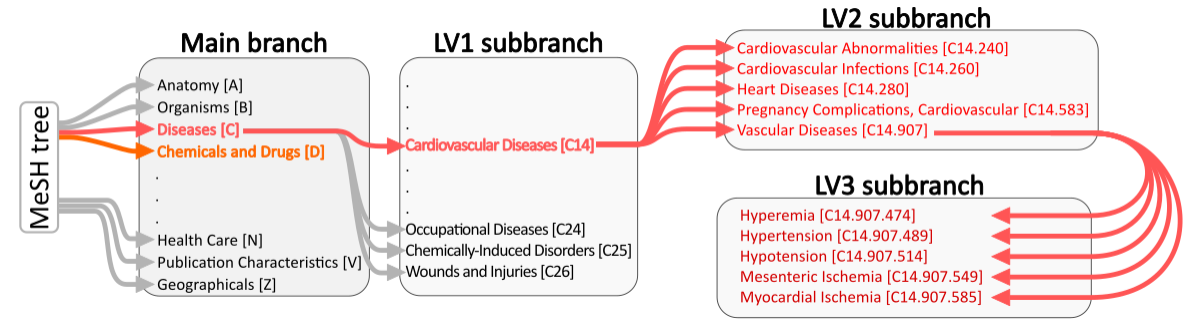
A schematic representation of the hierarchical structure within the MeSH (Medical Subject Headings) tree, illustrating the organization from main branches to more specific sub-branches. The main branch example shown here includes categories like Anatomy [A], Diseases [C], and Chemicals and Drugs [D]. It details the progression from a main branch (Diseases [C]) to a LV1 sub-branch (Cardiovascular Diseases [C14]), to more refined LV2 and LV3 sub-branches, which specify narrower topics such as Heart Diseases [C14.280] and further down to Hypertension [C14.907.489] within the LV3 sub-branch. Each descriptor or topic is paired with a unique alphanumeric code that facilitates indexing and retrieval in medical databases. LV1: level 1; LV2: level 2; LV3: level 3.

In the MeSH tree, the 16 main categories form the foundation of its hierarchical structure. Each main category branches into level 1 (LV1) subbranches, representing more specific aspects of the primary category. These LV1 subbranches further divide into level 2 (LV2) subbranches, offering an even more detailed classification. This pattern continues, with each subsequent level—level 3 (LV3), level 4, and so forth—delving deeper into specialized topics, ensuring a comprehensive and nuanced organization of medical subjects. Overall, the MeSH descriptors are structured hierarchically across 13 levels of subbranches. The coding of MeSH descriptors involves assigning unique alphanumeric identifiers to each descriptor in the MeSH database. These codes serve as precise references, facilitating information retrieval and classification in medical and health-related databases. Typically, MeSH codes consist of a combination of letters and numbers. The letters often represent the main category or aspect of health or medicine the descriptor pertains to, while the numbers provide a unique identifier within that category. Figure 1 presents a schematic representation of the MeSH tree.

For our analysis, we developed a Python script capable of mining relevant publications from PubMed through their API. It extracts the MeSH descriptors associated with an article, along with its unique PMID, and translates a given MeSH descriptor code to the corresponding MeSH descriptor name. Since the code of the MeSH descriptor embeds the location of the term in the MeSH tree, our script can determine the branches from which a MeSH descriptor originates. Our analysis primarily focuses on the “Diseases” main branch (denoted by the letter C), especially the “Cardiovascular Diseases” subcategory (LV1 subbranch C14; Figure 1). The hierarchical structure of the MeSH tree enables an in-depth analysis of topics at different levels of specificity, as illustrated in Figure 1. A more detailed description of the algorithm is given in the subsequent sections.

### Creation of the Database

**Figure 2:**
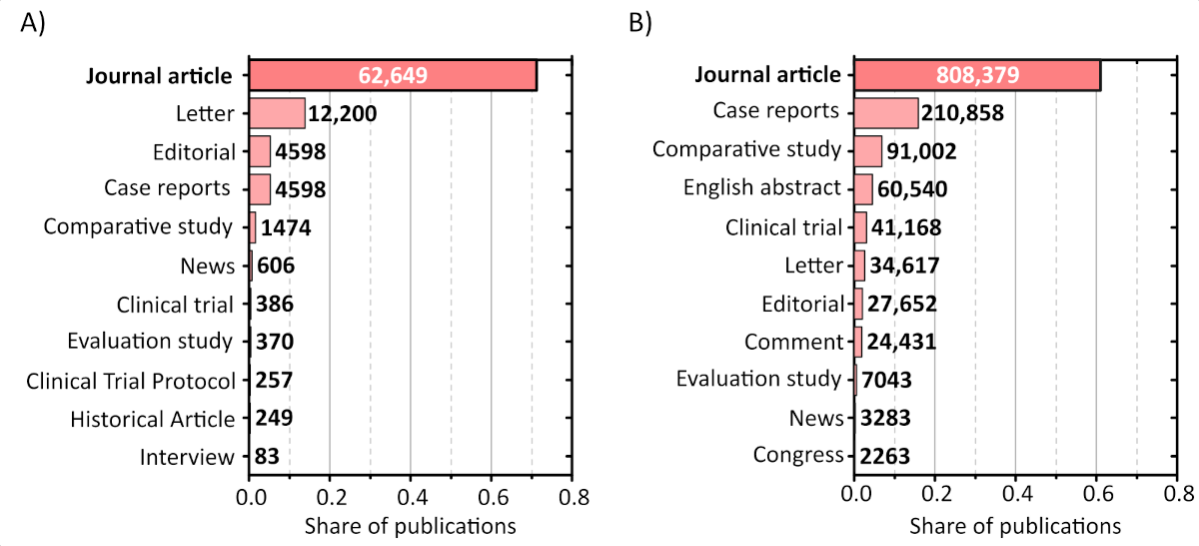
Publication type prevalence in the (A) COVID-19 and (B) cardiovascular disease (CVD) data sets.

Using Python and the PubMed API, Entrez, our algorithm retrieved relevant information from the PubMed database on COVID-19 and CVDs. We utilized MeSH descriptors as search parameters. For the CVD database, our search query was “Cardiovascular Diseases [MeSH Terms],” while for the COVID-19 database, it was “COVID-19 [MeSH Terms].” Our inclusion criteria were limited to articles from peer-reviewed journals. Figure 2 provides a comprehensive breakdown of the records obtained for each query, categorized by publication type, focusing on the PMID and associated MeSH terms.

For the selected publications, we retrieved raw XML data from PubMed and extracted 2 pieces of information from each XML file: all MeSH descriptors and the PMID. The latter was used to remove duplicate entries. Following this procedure, we created 2 databases: one for the COVID-19 query and another for the CVD query. In addition to these databases, we also developed a graph of MeSH descriptors, which was used for information retrieval.

### Data Analysis

For each database, we performed statistical analyses using Python [46] and its associated libraries: pandas [47] for data manipulation, SciPy [48] for statistical calculations, and Matplotlib [49] for data visualization.

Our initial step in the analysis involved calculating the relative frequency of each MeSH descriptor within a specified branch level, ranging from the “Disease” main branch to subsequent levels such as the LV1 subbranch and beyond. We accomplished this by tallying the occurrences of each MeSH term across all publications in our database. After obtaining these raw counts, we moved to a critical phase of normalization. We normalized each count by the total number of articles within the database, thus converting raw frequencies into proportional measures. This adjustment allows the data to accurately reflect the prevalence of each descriptor within the context of the overall literature corpus.

For a more granular analysis of specific subbranches (i.e., LV1, LV2, etc), we refined our approach. We quantified the number of articles associated with each MeSH descriptor within the subbranch of interest. This time, however, the normalization process took into account the total number of articles relevant to that particular subbranch, thus ensuring that our statistical insights were accurately contextualized within the scope of the subbranch’s literature.

To ascertain the relative significance of specific MeSH descriptors within our databases, we denoted the frequency of each MeSH term within a database (DB) as *f*_DB_ (MeSH). This measure allows us to conduct a comparative analysis to determine the prominence of each descriptor in the COVID-19 database relative to the CVD database. We measure the disparity in usage frequency of a MeSH term between the 2 databases by calculating the difference , expressed as:

Δ*f*(*MeSH*) = *f*_COVID_(*MeSH*) – *f*_CVD_(*MeSH*) **(1)**

This difference, Δ*f*(*MeSH*), provides an indication of whether a MeSH descriptor’s presence is more pronounced (up-regulated) or less pronounced (down-regulated) in the COVID-19 database as compared to the CVD database. A positive difference signifies a MeSH term’s greater relevance to the COVID-19 corpus, while a negative value indicates lesser importance.

However, the difference in frequencies can be misleading if the absolute values are too large or small. This difference might not accurately represent the term’s practical significance. To address this, we also calculated the ratio, *R*(*MeSH*), defined as:




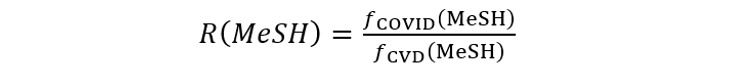

**(2)**



This ratio offers insight into the relative usage of each MeSH descriptor. A ratio near 1 suggests comparable usage in both databases, while ratios significantly greater or less than 1 imply a disparity in descriptor usage.

By integrating both the difference, Δ*f*(*MeSH*), and the ratio, *R*(*MeSH*), of frequencies, we achieved a more nuanced understanding of the role and emphasis of MeSH terms in the COVID-19 database in contrast with the CVD database. This dual-parameter approach allows for a more detailed and representative interpretation of the importance of specific MeSH descriptors in relation to the topics under investigation, such as hypertension.

### Statistical Analysis

To pinpoint the most significant MeSH topics within the context of the 3 most prominent CVDs in relation to COVID-19, we employed a statistical approach. We conducted a chi-square test of independence. This statistical test was employed to assess whether the occurrence of specific MeSH terms shows a significant difference when comparing the COVID-19 database with the CVD database.

The chi-square test is particularly suited for this analysis as it helps determine if there is a significant association between the type of database (COVID-19 or CVD) and the frequency of particular MeSH terms. A significant result from this test implies that the likelihood of a MeSH term’s occurrence is dependent on the database, indicating a specific relevance to either COVID-19 or CVD-related articles.

Such a methodological approach allows us to identify and highlight those MeSH terms that are disproportionately represented in one database compared to the other, thereby providing insights into the intersection of COVID-19 with prominent cardiovascular conditions. This analysis not only enhances our understanding of disease dynamics but also potentially guides future research directions in these intersecting medical areas.

## Results

Our analysis begins with a thorough examination of the “Disease” main branch in the MeSH tree. Specifically, our interest lies in the corresponding MeSH descriptors found within the LV1 subbranches. These LV1 subbranches are particularly notable as they encompass the primary disease descriptors, which are the fundamental classifications for various diseases.

**Figure 3:**
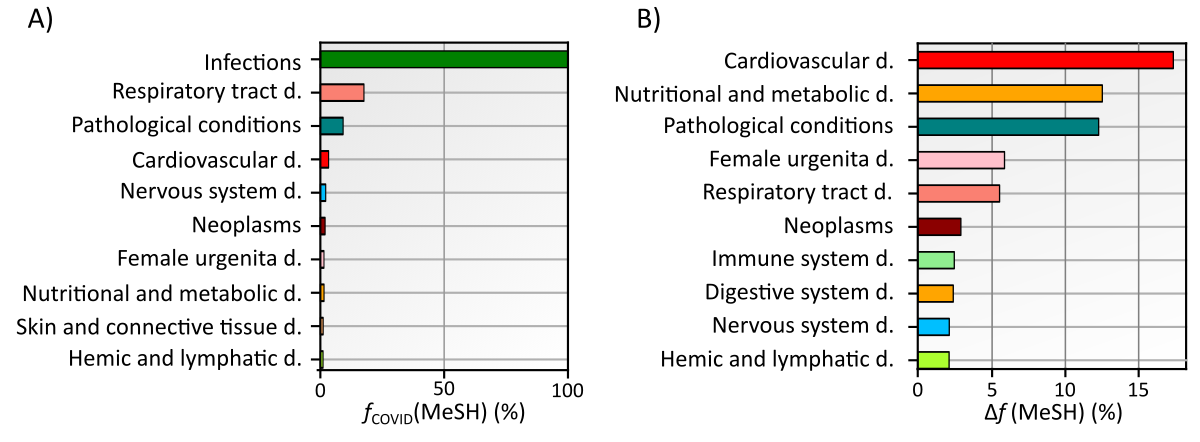
Comparative analysis of level 1 (LV1) subbranch disease descriptor frequencies in COVID-19–related articles. Panel A presents the distribution within the COVID-19 data set, while Panel B focuses on the subset of COVID-19 articles tagged with the “Comorbidity” MeSH (Medical Subject Headings) term. Each bar’s color corresponds to a specific disease descriptor and maintains consistency throughout the manuscript.

In Figure 3, we show the frequency with which primary disease descriptors are used in COVID-19 articles, *f*_COVID_(*MeSH*). Additionally, we analyze articles that have been assigned the MeSH term “Comorbidity,” focusing exclusively on the frequency of disease descriptors within this subset. We compute the relative importance as defined in equation (1) to assess the importance of each LV1 disease descriptor in the context of comorbidity-related articles.

Overall, we find that all COVID-19 articles are labeled with the LV1 disease descriptor “Infections.” The second most common LV1 disease descriptor is “Respiratory Diseases,” which appears in 26.7% of all articles. The third descriptor, “Pathological Conditions, Signs and Symptoms,” was found in about 20% of all COVID-19 articles. The MeSH term “Cardiovascular Diseases” is the fourth most used descriptor, found in 7% of all articles. The top ten LV1 disease descriptors found in COVID-19 articles are shown in Figure 3A. In contrast, the results in Figure 3B illustrate the relative importance of the disease descriptors in a subset of COVID-19 articles related to comorbidities, considering the baseline frequency shown in Figure 3A. Therefore, the results in Figure 3B evaluate the importance of each LV1 disease descriptor specifically for these selected articles. As can be seen in Figure 3B, “Cardiovascular Diseases” has the highest relative importance among LV1 disease descriptors in COVID-19 articles examining comorbidity. This MeSH term has a 17.4% higher frequency of occurrence among COVID-19 articles related to comorbidities. It is also interesting to note that the MeSH term “Nutritional and Metabolic Diseases” ranks second.

**Figure 4:**
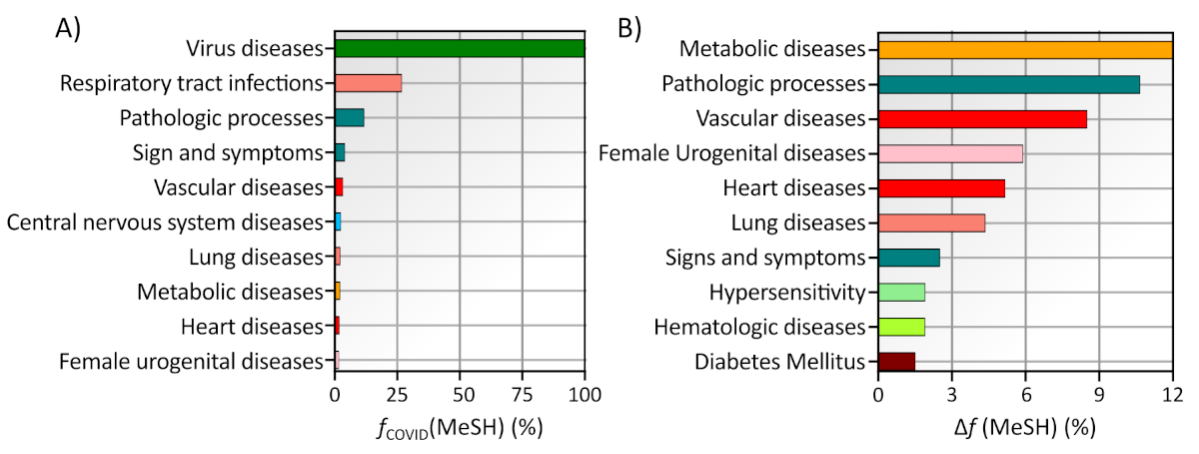
Most important level 2 (LV2) disease descriptor. Results are computed for (A) the entire COVID-19 data set and (B) for the subset of COVID-19 articles related to comorbidities. Each bar’s color corresponds to a specific disease descriptor, as defined in Figure 3. MeSH: Medical Subject Headings.

In continuation, we focus on the first- and second-ranked MeSH descriptors in Figure 3B. To describe the disease terms in more detail, we repeat the analysis at the second level of the MeSH disease tree. Again, we separately calculated the proportion of items with a given LV2 disease descriptor and the relative importance of these descriptors within COVID-19 articles related to comorbidities. The results are shown in Figure 4.

**Figure 5:**
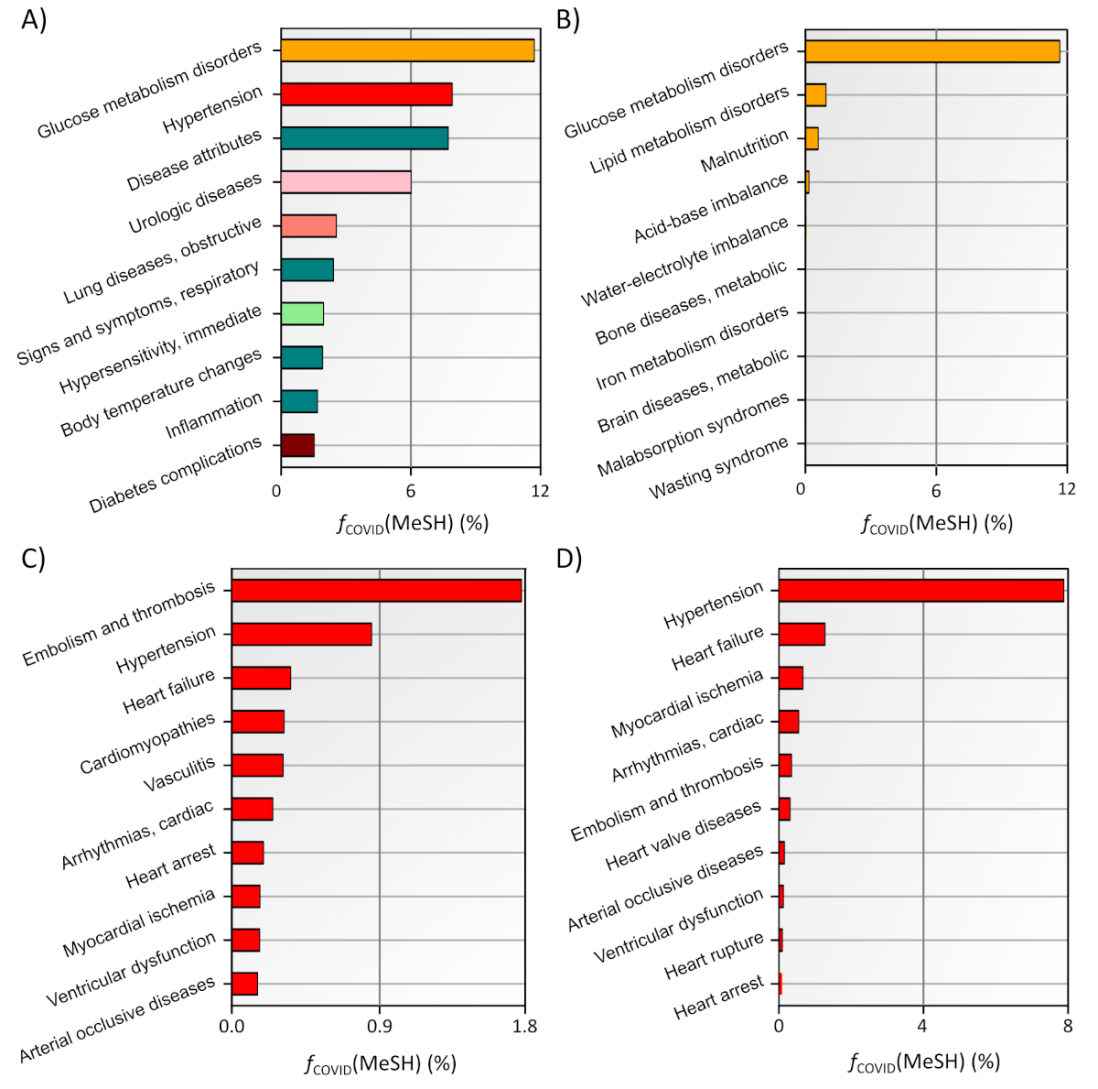
Ten most important level 3 (LV3) disease descriptors for COVID-19–related articles. Results are computed for (A) all LV3 disease descriptors, (B) only for LV3 disease descriptors originating from the “Nutritional and Metabolic Diseases,” (C) only for the LV3 “Cardiovascular Diseases” branch, and (D) only for the LV3 “Cardiovascular Diseases” branch obtained for the COVID-19 sub-set of articles considering comorbidities. Each bar’s color corresponds to a specific disease descriptor, as defined in Figure 3. MeSH: Medical Subject Headings.

From the results shown in Figure 4A, we see that “vascular disease“ and “Heart Disease,” which belong to “Cardiovascular Diseases,” are among the 10 most frequently used LV2 disease descriptors. For COVID-19 articles related to comorbidity, both “Vascular Disease” and “Heart Disease” gain prominence (Figure 4B). The LV1 subbranch “Cardiovascular Diseases” is divided into 5 MeSH descriptors at the second level (Cardiovascular Abnormalities, Cardiovascular Infections, Heart Diseases, Pregnancy Complications, Cardiovascular and Vascular Diseases). In contrast, the disease branch “Nutritional and Metabolic Diseases” is divided into 2 descriptors at the second level (Metabolic Diseases, Nutritional Disorders). This should be considered as it could lead to a bias in the frequency of occurrence of a descriptor caused by the number of terms available in each subbranch. However, since we are interested in their relative importance, we can circumvent these biases and reveal the distributed or concentrated importance of the descriptors. Therefore, we continue our analysis at the third level of the MeSH tree of diseases. Since we found that at the second level of the MeSH tree, the LV1 subbranch “Cardiovascular Diseases” and “Nutritional and Metabolic Diseases” have the highest relative importance, we continue our investigation in this direction. The results are shown in Figure 5.

Figure 5A reveals that “Disorders of Glucose Metabolism” top the list as the most frequently mentioned LV3 MeSH term within the COVID-19 data set, followed by “Hypertension” and “Disease Attributes.” This figure provides an overarching view of the commonality of these terms across all research articles.

Figure 5B delves into the LV3 MeSH descriptors that stem from the LV2 subbranch of “Metabolic Diseases”. The data clearly indicate that disorders of glucose and lipid metabolism are the most recurrent topics within the LV3 subbranch, underscoring their significance in the discourse on metabolic diseases.

Figure 5C illustrates that within the realm of CVDs, “Embolism and Thrombosis” emerges as the most prevalent LV3 MeSH descriptor utilized in the literature, followed by “Hypertension” and “Heart Failure,” among others, in descending order of frequency.

By comparing Figure 5B and 5C, we observe a less diverse distribution of the embedded MeSH terms. The LV3 descriptors within the CVD subbranch are more specifically clustered, pointing to a narrower focus within CVD research in relation to COVID-19, as opposed to the broader range of topics covered under metabolic diseases.

Figure 5D presents a detailed ranking of LV3 MeSH descriptors within the CVD domain as they appear in the context of comorbidity research. Figure 5D specifically highlights which cardiovascular conditions are most frequently discussed in conjunction with other health issues, shedding light on the patterns of comorbidity that are prevalent in the current body of literature. It allows researchers to identify which cardiovascular disorders are most considered in studies that address the complexities of patients presenting with multiple concurrent health challenges. The data presented in Figure 5D identifies “Hypertension” as the most used LV3 MeSH descriptor within articles that discuss comorbidities, with “Heart Failure” following in frequency. Based on these observations from Figure 5, the subsequent analysis concentrates on 3 critical LV3 MeSH terms: “Hypertension,” “Heart Failure,” and “Embolism and Thrombosis.”

**Figure 6:**
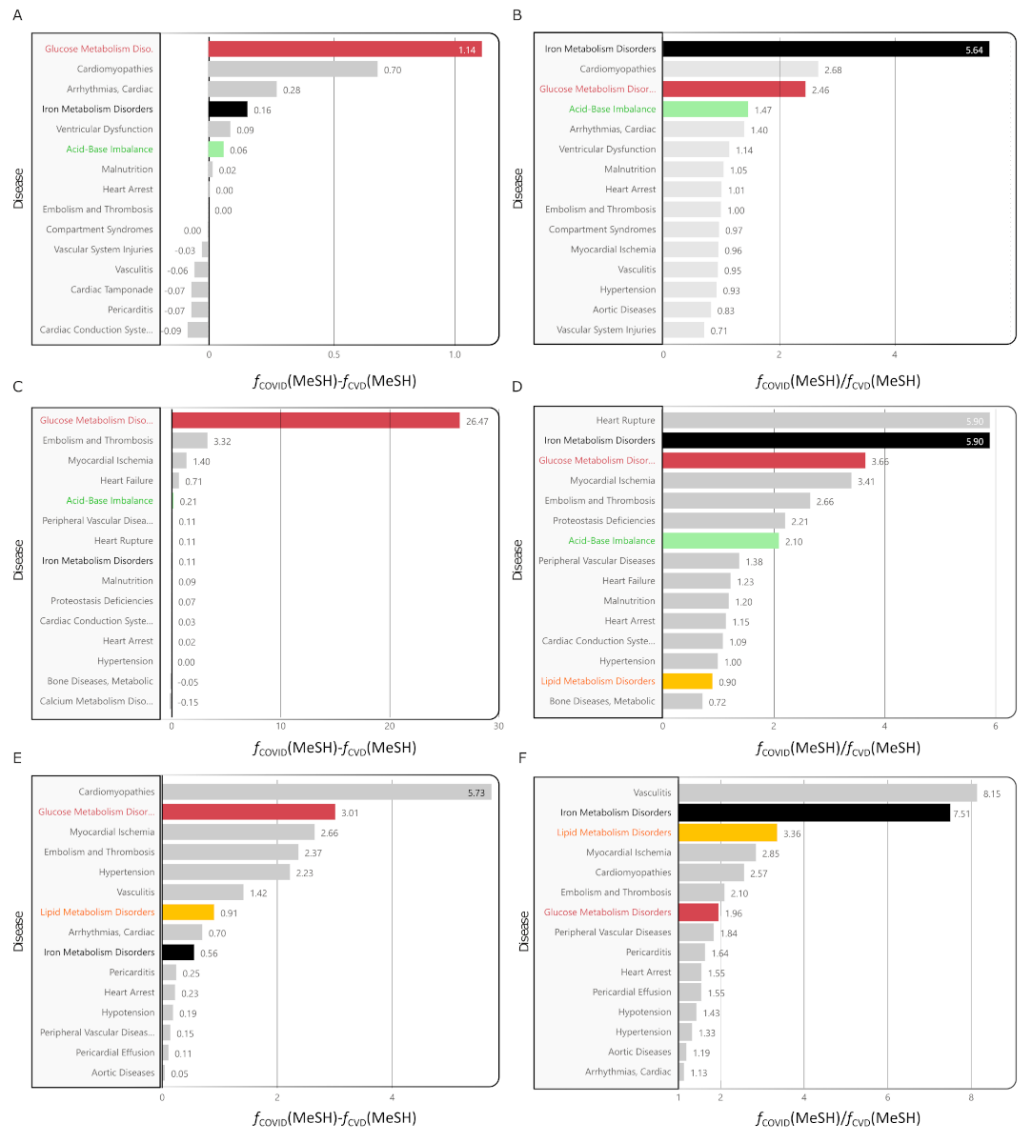
A comprehensive comparison of the absolute and relative changes in the frequency of level 3 (LV3) MeSH (Medical Subject Headings) descriptors in the COVID-19 database relative to the cardiovascular disease (CVD) database, focusing on 3 specific cardiovascular diseases: Embolism and Thrombosis (panels A and B), Hypertension (panels C and D), and Heart Failure (panels E and F). The figure displays the top 15 MeSH descriptors for each condition. Notably, the bars corresponding to “Iron Metabolism Disorders,” “Glucose Metabolic Disorders,” “Acid-Base Imbalance,” and “Disorders of Lipid Metabolism” are distinctly color-coded in black, red, green, and orange, respectively, allowing for easy identification and comparison of these key terms across different cardiovascular conditions.

In our analysis of the COVID-19 and CVD databases, we use the frequency of LV3 MeSH descriptors to represent the focus of research. We started by examining “Embolism and Thrombosis,” a common CVD descriptor (Figure 5C). Our results (Figure 6A) indicate that “Embolism and Thrombosis” is most frequently associated with “Disorders of Glucose Metabolism” in the COVID-19 database. “Disorders of Iron Metabolism” (with an increase of ∆*f*[MeSH]=0.16% and a ratio of *R*[MeSH]=5.64) and “Disorders of Acid-Base Balance” are also significant but less frequent. “Disorders of Iron Metabolism” have seen the largest increase, ranking it at the top in the COVID-19 database. “Disorders of Glucose Metabolism” follow (with an increase of ∆*f*[MeSH]=1.14% and a ratio of *R*[MeSH]=2.46), and “Disorders of Acid-Base Balance” come in third (with an increase of ∆*f*[MeSH]=0.06% and a ratio of *R*[MeSH]=1.47).

For high blood pressure (“Hypertension”), the MeSH term “Disorders of Glucose Metabolism” is most significant in frequency difference, followed by “Acid-Base Imbalance” and “Iron Metabolism Disorders” (Figure 6C). Interestingly, “Iron Metabolism Disorders” show a smaller overall frequency difference (∆*f*[MeSH]=0.11%) but a higher ratio (*R*[MeSH]=5.90), indicating they are used more frequently in COVID-19 research compared to CVD research. “Disorders of Glucose Metabolism” take the second spot (with a substantial increase of ∆*f*[MeSH]=26.47% and a ratio of *R*[MeSH]=3.66), and “Acid-Base Imbalance” is third (∆*f*[MeSH]=0.21% and *R*[MeSH]=2.10) based on their relative frequencies.

In the third part of our analysis, we focused on heart failure, with the findings illustrated in Figure 6E and 6F. Among the various MeSH terms, “Glucose Metabolic Disorders” emerged as the second most frequent term in the comparison between the COVID-19 and CVD databases. While “Iron Metabolic Disorders” and “Acid-Base Imbalance” are also relevant, they are positioned at 17th, explaining their absence from the figure due to their lower frequency. Notably, “Iron Metabolism Disorders” feature more prominently in the COVID-19 database than in the CVD database, ranking tenth in frequency difference. Significantly, as Figure 6F reveals, “Iron Metabolism Disorders” rank second in relative importance among all LV3 MeSH descriptors in the COVID-19 database, compared to the CVD database. “Disorders of Lipid Metabolism” also show considerable relevance, ranking third, whereas “Disorders of Glucose Metabolism” are positioned seventh. Despite its lower frequency, “Acid-Base Imbalance” maintains a high relative importance, coming in at 17th. These results underscore the shifted focus in medical research on specific metabolic disorders in the context of COVID-19, particularly in relation to heart failure.

To build upon these findings, we applied the chi-square test to validate whether the observed differences in LV3 MeSH descriptor frequencies between COVID-19 and CVD databases are statistically significant. This test helped us determine if the occurrences of 4 specific MeSH terms—“Disorders of Glucose Metabolism,” “Iron Metabolism Disorders,” “Acid-Base Imbalance,” and “Disorders of Lipid Metabolism”—in the COVID-19 database are significantly different from their occurrences in the CVD database. We also employed multiple *P* values to strengthen our assessment of significance. The findings are detailed in Table 1.

**Table 1:** Statistical significance of selected MeSH (Medical Subject Headings) terms in 3 subsets of COVID-19 articles related to cardiovascular diseases (CVDs)^a^.

LV3^b^ MeSH terms	Embolism and thrombosis, *P* value	Hypertension, *P* value	Heart failure, *P* value
Glucose Metabolism Disorders	<.001	<.001	<.05
Iron Metabolism Disorders	<.01	.525	.116
Acid-Base Imbalance	.103	.42	.585
Lipid Metabolism Disorders	.94	.53	.05

^a^The *P* values signify whether the appearance of a MeSH term in the COVID-19 database is significantly different compared to the appearance in the CVD database.

^b^LV3: level 3.

In the context of “Embolism and Thrombosis,” our analysis reveals that the frequencies of both glucose and iron metabolism disorders show a statistically significant difference when comparing the COVID-19 and CVD databases across all 3 subdata sets.

For “Hypertension,” the scenario is slightly different. Here, the incidence of glucose metabolism disorders stands out as the only descriptor with a significant difference in frequency between the COVID-19 and CVD databases.

Lastly, regarding “Heart Failure,” we again note a significant difference for glucose metabolism disorders, although with a *P* value of <.05. This pattern highlights a specific focus or heightened research interest in glucose metabolism disorders within the context of COVID-19, particularly when comorbid with CVDs such as embolism, thrombosis, hypertension, and heart failure.

## Discussion

### Principal Results

The aim of this study was to analyze all available peer-reviewed articles from the PubMed database to identify the most relevant topics regarding the relationship among COVID-19, CVDs, and comorbidity. For this purpose, we used the MeSH term descriptors, which are the most important topics covered in an article in a standardized form. In COVID-19–related research that considers comorbidity, we found the most relevant MeSH descriptors are CVDs and nutritional and metabolic diseases. Since both terms are quite broad, we continued our analysis one branch deeper in the MeSH tree and found that the corresponding significant topics are related to metabolic disorders, vascular diseases, and heart diseases. Advancing one level deeper in the MeSH tree, we investigated the meaning of more specific terms related to CVD and metabolic disease. We determined that the most significant CVDs related to comorbidity and COVID-19 are embolism and thrombosis, hypertension, and heart failure. Given the prominence of metabolic disorders in our analysis, we also explored which specific metabolic disorders were most significant and found that glucose metabolism disorders were the most notable. However, we also noted a significantly increased frequency of the term iron metabolism disorders in COVID-19 articles related to embolism and thrombosis compared to CVD articles related to embolism and thrombosis.

### Limitations

Using the methodology presented here, we were able to identify the most important issues relevant to comorbidities and COVID-19. Although the methodology can be applied to any major topic and its corresponding subtopic, it has some limitations. The main limitation is its inability to find relationships between themes. This was addressed by selecting relevant subtopics through iteratively evaluating the results at each level of the MeSH tree. However, in future studies, we intend to incorporate a knowledge graph-based approach by mapping relationships between topics. This would in turn allow us to consider not only the frequency of the occurrence of a topic but also to evaluate the co-occurrence of topics. Consequently, this would allow us to automatically find highly related pairs of topics and eventually create a more detailed and complex description of the item database under consideration.

### Comparison With Prior Work

In relation to COVID-19, individuals with certain comorbidities have been shown to have a higher likelihood of developing a severe form of this disease and have a higher mortality rate. COVID-19 has been associated with an increased prevalence of CVD, suggesting that CVD may be a risk factor for the disease [50]. According to mortality data from China’s National Health Commission, 17% and 35% of individuals with COVID-19 had a history of coronary heart disease and hypertension, respectively [51]. Li et al [52] showed that the presence of cardio-cerebrovascular disease, diabetes, and hypertension increased the risk of severe COVID-19 by threefold, twofold, and twofold, respectively. A larger study from the Chinese Center for Disease Control and Prevention, which examined the clinical outcomes of 44,672 confirmed COVID-19 cases, found that the case fatality rate was 2.3% in the entire cohort, but was significantly higher (6%, 7.3%, and 10.5%, respectively) in individuals with hypertension, diabetes, and CVD [53]. Several smaller cohort studies have also presented similar reports, suggesting a higher risk of an adverse episode in patients with COVID-19 with underlying CVD [54-56]. Cardiac injury (characterized by elevated troponin levels), myocarditis, and acute respiratory distress syndrome have been reported as strong, independent risk factors associated with mortality in patients with COVID-19 [57]. According to the Pneumonitis Diagnosis and Treatment Program for Novel Coronavirus Infections, the likelihood of COVID-19 infection is higher in older people (>60 years) with pre-existing conditions, especially in patients with hypertension, coronary heart disease, or diabetes [51]. Thus, advanced age, male gender, and the presence of preexisting conditions are the main risk factors for COVID-19 mortality [57].

Given the increasing evidence of iron status’ importance for immunity, it is not surprising that biomarkers of iron metabolism have been investigated in several studies on patients with COVID-19 [58]. COVID-19 is also characterized by a cytokine storm, leading to increased production of hepcidin, the primary hormone regulating iron metabolism, in response to heightened proinflammatory cytokines [59]. Patients with low serum iron status were likely to suffer from severe conditions and multiple organ damage in COVID-19 [60]. In addition, both iron deficiency (ID) and iron overload are commonly observed in a variety of CVDs and contribute to the onset and progression of these diseases. One of the devastating consequences of iron overload is the induction of ferroptosis, a newly defined form of regulated cell death that severely impairs cardiac function through ferroptotic cell death in cardiomyocytes [60]. Our results show that the term iron metabolism disorder occurs significantly more frequently in COVID-19 articles related to heart failure than in CVD articles on the same topic. Interestingly, ID is frequently observed in patients with heart failure [61-63]. Furthermore, ID correlates with an increased incidence of right ventricular failure in patients with acute HF [64,65]. ID also contributes to impaired functioning of the respiratory chain complexes (complex I to V), leading to altered myocardial metabolism, ROS formation, and ultimately advanced HF. Impaired mitochondrial function is one of the underlying mechanisms of ID-induced HF [66,67].

### Clinical Implications of Our Findings

Our research has highlighted the critical intersection between COVID-19 and severe cardiovascular conditions, notably embolism and thrombosis. The urgency of identifying and managing these conditions is of paramount importance, as they present immediate life-threatening risks and their symptoms often overlap with those of COVID-19, especially pulmonary thromboembolism [68]. Our findings underscore the vital importance of vigilant monitoring for individuals affected by COVID-19 to prevent these severe outcomes.

A primary tool in this monitoring process is the serial measurement of D-dimer levels, which has been shown to strongly correlate with an increased risk of disease progression, critical illness, and mortality. D-dimer levels also serve as a reliable predictor of venous thromboembolism when measured at admission, and levels at discharge are associated with a higher 1-year mortality risk [69]. Current guidelines recommend thromboprophylaxis for all hospitalized patients with COVID-19, except those with an increased risk of bleeding [68]. While further research is necessary to determine the optimal anticoagulation dosage, standard doses of LMWH are generally recommended for most patients, with intermediate doses for those who are critically ill or obese [70]. Routine screening for deep vein thrombosis with Doppler ultrasonography is not currently advised for thromboembolism screening, as rapidly increasing D-dimer levels and worsening oxygenation have been found to be more successful [71].

We have also uncovered a significant correlation between COVID-19 and glucose metabolism disorders. Increasing evidence suggests a bidirectional relationship between diabetes and SARS-CoV-2 infection. This indicates that patients with diabetes are at a higher risk of developing a severe form of COVID-19, while individuals with COVID-19 are more likely to develop metabolic disorders. Shared pathogenic mechanisms, such as general inflammation, a pro-thrombotic state, and atherosclerosis, likely contribute to this association [72].

Analysis of the GTEx database revealed higher ACE2 expression in the pancreas than in the lungs. Liu et al. analyzed pancreatic injury following SARS-CoV-2 infection and found that such injuries predominantly occurred in patients with severe COVID-19 [73]. Therefore, special attention is warranted for patients with metabolic disorders, including priority for vaccination and rigorous monitoring in the event of infection, with a low threshold for intensifying care. Preventive measures for detecting metabolic disorders should be implemented in individuals after a severe SARS-CoV-2 infection. This includes monitoring blood glucose levels, lipids, and biochemical markers for pancreatic injury.

Additionally, our results underscore the importance of iron metabolism, a factor currently underrepresented in clinical practice, underscoring the need for further trials to integrate it into care for patients with COVID-19. Research indicates that ferritin levels can be used to estimate disease severity, providing useful cutoff values. These could complement other initial screening methods in predicting the necessary level and intensity of patient care [74]. There is also an underexplored therapeutic potential in manipulating iron levels, either by using chelators like deferoxamine to lower them or through iron supplementation to raise them in patients with inappropriate values. Before this approach can be widely adopted in practice, further research is essential to determine the optimal levels. Reducing iron in patients with highly active hepcidin due to inflammation could impede recovery [75]. Nonetheless, the importance of iron metabolism extends beyond coagulation disorders to metabolic disorders, with iron overload contributing to the development of these diseases [76,77].

In summary, the insights from our study have critical implications for clinical practice. By identifying key biomarkers and conditions associated with severe COVID-19 outcomes, we provide a foundation for improving patient monitoring, treatment strategies, and, ultimately, patient outcomes during the pandemic. Our findings urge health care professionals to incorporate these insights into their clinical practice, promoting a proactive and informed approach to managing COVID-19 and its cardiovascular complications.

### Conclusions

Our study represents a crucial step toward understanding the complex interplay between COVID-19, CVD, and metabolic disorders, highlighting in particular the role of embolism, thrombosis, and iron metabolism disorders. The method we adopted, using MeSH term descriptors to dissect the different levels of related topics, has furnished a comprehensive overview of the main comorbidities influencing COVID-19 outcomes. Importantly, this approach can be adapted and applied to other important health topics and their subcategories, despite its current limitation in directly mapping topic relationships. Future research efforts should aim to incorporate a knowledge graph–based methodology, enabling a more detailed analysis of topic co-occurrences and their relationships. Such advancements are essential for deciphering complex disease dynamics, particularly in the context of emerging infectious diseases such as COVID-19.

The knowledge gained from this study is invaluable for the development of more effective clinical practices and public health strategies. By identifying key comorbidities and their impact on COVID-19, we are better positioned to tailor treatments and interventions for patients affected by these conditions. Additionally, understanding the role of specific metabolic disorders, such as those affecting glucose and iron metabolism, opens up potential therapeutic targets and preventive measures. In managing the current pandemic and preparing for future viral outbreaks, the findings from this study are crucial in guiding medical advancements, improving patient outcomes, and increasing the resilience of the health care system. This work not only contributes to our immediate fight against COVID-19 but also creates a foundation for more informed and effective responses to similar health crises in the future.

## Abbreviations

ACE2: angiotensin-converting enzyme 2
CVD: cardiovascular disease
DB: database
ID: iron deficiency
LV1: level 1
LV2: level 2
LV3: level 3
MeSH: Medical Subject Headings
NLM: National Library of Medicine
PMID: PubMed identifier
RAAS: renin-angiotensin-aldosterone system
